# Cellulose-Cyclodextrin Co-Polymer for the Removal of Cyanotoxins on Water Sources

**DOI:** 10.3390/polym11122075

**Published:** 2019-12-12

**Authors:** Diego Gomez-Maldonado, Iris Beatriz Vega Erramuspe, Ilari Filpponen, Leena-Sisko Johansson, Salvatore Lombardo, Junyong Zhu, Wim Thielemans, Maria S. Peresin

**Affiliations:** 1 Forest Products Development Center, School of Forestry and Wildlife Science, Auburn University, 520 Devall Drive, Auburn, AL 36830, USA; dzg0023@auburn.edu (D.G.-M.); ibv0002@auburn.edu (I.B.V.E.); ilari.filpponen@auburn.edu (I.F.); 2 Department of Chemical Engineering, Alabama Center for Paper and Bioresource Engineering (AC-PABE), Auburn University, 358 Ross Hall, Auburn, AL 36849, USA; 3 Department of Bioprocesses and Biosystems, Aalto School of Chemical Technology, BIO2, P.O. Box 16100, 02150 Espoo, Finland; leena-sisko.johansson@aalto.fi; 4 Renewable Materials and Nanotechnology Research Group, Department of Chemical Engineering, KU Leuven, Campus Kulka Kortrijk, Etienne Sabbelaan 53, 8500 Kortrijk, Belgium; salvatore.lombardo@kuleuven.be (S.L.); wim.thielemans@kuleuven.be (W.T.); 5 USDA Forest Products Laboratory, 1 Gifford Pinchot, Madison, WI 53726, USA; junyong.zhu@usda.gov

**Keywords:** bio-based composite, cellulose nanofibrils, β-cyclodextrin, surface chemistry, water treatment, cyanotoxins, microcystin-LR

## Abstract

With increasing global water temperatures and nutrient runoff in recent decades, the blooming season of algae lasts longer, resulting in toxin concentrations that exceed safe limits for human consumption and for recreational use. From the different toxins, microcystin-LR has been reported as the main cyanotoxin related to liver cancer, and consequently its abundance in water is constantly monitored. In this work, we report a methodology for decorating cellulose nanofibrils with β-cyclodextrin or with poly(β-cyclodextrin) which were tested for the recovery of microcystin from synthetic water. The adsorption was followed by Quartz Crystal Microbalance with Dissipation monitoring (QCM-D), allowing for real-time monitoring of the adsorption behavior. A maximum recovery of 196 mg/g was obtained with the modified by cyclodextrin. Characterization of the modified substrate was confirmed with Fourier Transform Infrared Spectroscopy (FT-IR), X-ray Photoelectron Spectroscopy (XPS), Thermogravimetric Analysis (TGA), and Atomic Force Microscopy (AFM).

## 1. Introduction

The demand for green materials has led to an increase in the development of materials derived from natural sources [1,2], especially when adsorption and removal of pollutants is the target application. This natural adsorbent can vary from composites derived from whole organisms such as *Aspergillus* [3], to more selective composites based on biopolymers, such as chitosan [4], or the more abundant cellulose. This latter one is the main component of cell wall structures of plants, algae, and a few other organisms. It therefore qualifies as the most abundant polysaccharide, making it interesting for utilization in renewable materials development [5,6,7]. In recent years, the increasing concerns over environmental changes and the decrease in water quality in recent years have led researchers from all around the world, to look for sustainable and low-cost materials that could be applied to improve water quality. Therefore, our interest is focused on eliminating of toxins that are a consequence of longer algae blooming seasons and cyanobacteria caused by an increasing temperature of water sources and nutrient runoff from agriculture [8,9,10,11].

Cellulose is a linear polysaccharide consisting of anhydroglucose units covalently linked by β-glycosidic bonds β-(1–4). These linear chains tend to form intra- and intermolecular hydrogen (H)-bonds to form bundles of fibrils with a hierarchical structure of varied diameters and lengths; the available ranges of dimensions and aspect ratio provide unique characteristics that can be utilized to design materials with the desired properties for the final use of the generated product. For example, cellulose nanofibrils (CNF), with diameters between 5–50 nm and lengths from hundreds of nanometers up to a few micrometers have a higher surface area than regular cellulose fibers. This in turn means that they have a larger amount of free hydroxyl groups on their surface which leads to a colloidal character and more reactive groups [11]. Also, the abundance of surface hydroxyl groups permits a variety of surface modifications either via chemical modification or physical adsorption. One common methodology is the surface modification to enable specific molecular adsorption, a preferred mechanism in water treatment [11,12,13,14].

**Figure 1:**
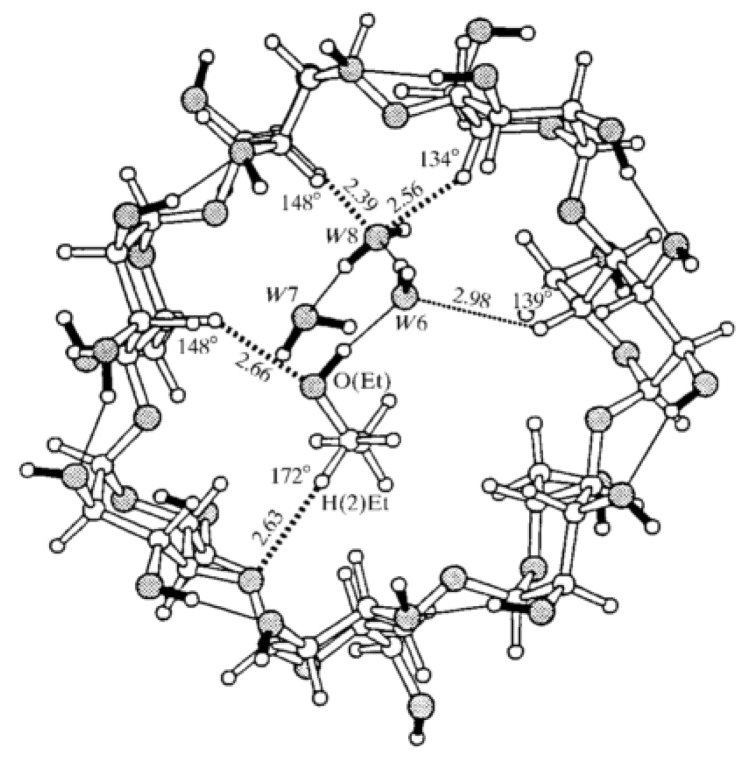
Neutron diffraction structure of β-cyclodextrin with ethanol complex. The hydrogen bonding capability can be observed through dash-lines. [17] Reproduced with the permission of International Union of Crystallography (https://journals.iucr.org/).

β-cyclodextrin has been demonstrated to effectively adsorb cyanotoxins [15,16] and it is also compatible with cellulose for producing composites. β-cyclodextrins are a seven-unit cone-shaped cycle of anhydroglucose units bonded by α-(1–4) bonds. The formation of this cone shape orients its hydroxyl groups of C2 and C3 into the wider extreme, leaving C6 and its hydroxyl group in the lower part and at the exterior of the cycle, generating a hydrophobic interior than can form inclusion complexes with a wide variety of molecules [17]. Figure 1 represents a neutron diffraction structure of this oligosaccharide and the bold black bars symbolize the hydroxyl groups of the structure. In the center, one can observe an entrapped ethanol molecule accompanied with three water molecules in the hydrophobic cavity of β-cyclodextrin: Thin solid lines represent the H-bonding between C2 and C3 of the anhydroglucose units, while the dotted lines illustrate the H-bonding with the inclusion molecule. The latter helps to capture and immobilize molecules which can be further occluded by hydrophobic interactions [18].

**Figure 2:**
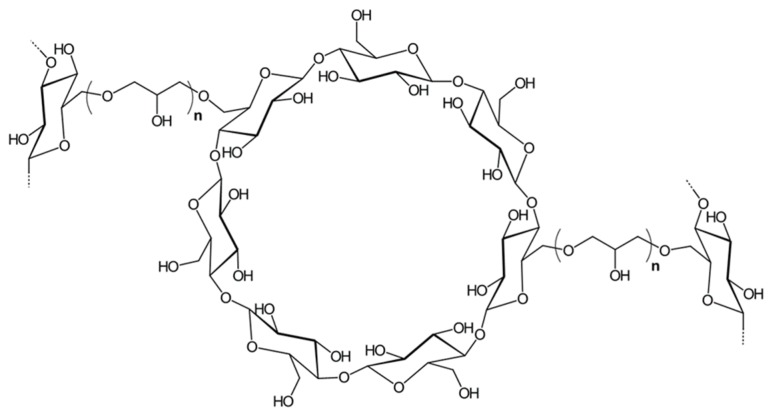
Expected structure of β-cyclodextrin crosslinked with other hydroxyl group from polysaccharides (cellulose or other cyclodextrins) through epichlorohydrin.

Grafting of cellulose with cyclodextrin has been demonstrated by different chemical methodologies, most of them based on the generation of a covalent bond between the hydroxyl groups of both components using linkers such as epichlorohydrin to form ether bonds [19,20,21,22,23,24]; or with poly(carboxylic) acids to form ester bonds [25,26,27]; however other possibilities with organic solvents, such as dimethyl sulfoxide, have also been achieved successfully [28]. The main operational differences between these two approaches are temperature and grafting medium. In particular, epichlorohydrin modification occurs in water at a mild temperature of 65 °C, while the esterification using polycarboxylic acids requires oven dry conditions at 180 °C. Moreover, oven drying has a significant downside when nanocellulose is utilized as a substrate, as fibers tend to aggregate due to hornification (H-bonding) as a consequence of losing the hydration layers which keep them separate. The expected configuration of β-cyclodextrin (CD) and its linkages through epichlorohydrin is schematically shown in Figure 2. This crosslinker introduces a new CH_2_ and ether linkages to stabilize the structure.

Among the different cyanotoxins, the most notable category is microcystins. These toxins are cyclic heptapeptides with varying amino acids at positions 2 and 4, which give them their particular names. In particular, microcystin-LR [29,30] must be constantly monitored as they have a proven connection to liver cancer. The World Health Organization (WHO) stipulates that the maximum concentration of microcystin-LR in water should not exceed 1 µg/L [31] but the United States’ Environmental Protection Agency (USEPA) [32] found out that up to 30% of the 1028 water sites tested had microcystin at concentrations between 0.1 µg/L to 225 µg/L, with an average of 3.0 µg/L, making this particular cyanotoxin one of the main targets to improve water quality.

**Figure 3:**
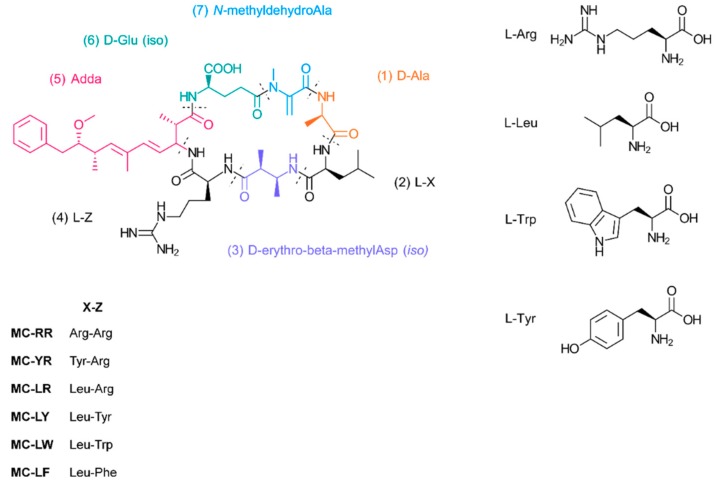
Basic chemical structure of microcystins. Amino acid residues 2 and 4 are interchangeable.

The basic chemical structure of microcystin is shown in Figure 3. As previously mentioned, amino acid residues 2 and 4 are interchangeable which alters the chemical properties of cyanotoxins. In addition, position 5 presents the amino acid 3-amino-9-methoxy-2,6,8-trimethyl-10-phenyldeca-4,6-dienoic acid (ADDA), which affords more hydrophobic character because of a long aliphatic chain and aromatic ring, and can therefore be exploited for the adsorption via hydrophobic interactions [30].

As previously mentioned, β-cyclodextrin has the tendency to form complexes with the hydrophobic molecules, especially with highly unsaturated structures such as benzene and its derivatives like benzocaine, as well as with amino acids such as tyrosine [18,33]. It has also been demonstrated that toxins containing ADDA are susceptible to form inclusion complexes with cyclodextrins driven by the hydrophobic interactions. These toxins include microcystin-LR, microcystin-RR, and nodularin-R [15,16].

Even though the mechanism of adsorption through the formation of complex β-cyclodextrin/microcystin-LR has been proven, the removal of the complex from water sources has remained a challenge. Different approaches such as magnetic particle carriers have been developed [16,34] but their sustainability and economic feasibility can be questioned. In this work, we aim to compare different configurations for the grafting of β-cyclodextrin onto cellulose nanofibrils as well as their ability to form inclusion complexes with microcystin-LR. To better understand the differences in the adsorption mechanisms, the generated materials were characterized by Fourier-Transform Infrared Spectroscopy with Attenuated Total Reflectance (FTIR-ATR), X-ray Photoelectron Spectroscopy (XPS), Atomic Force Microscopy (AFM), and Thermogravimetric Analysis (TGA); while the adsorption capacity was measured by Quartz Crystal Microbalance with Dissipation monitoring (QCM-D).

## 2. Materials and Methods

### 2.1. Materials

The base material, cellulose nanofibrils (CNF), was prepared by using concentrated di-carboxylic acid hydrolysis with subsequent mechanical fibrillation as described previously [35,36]. Then, 1 kg (oven dry) of bleached eucalyptus Kraft pulp fibers (BEP) from a commercial source (Aracruz Cellulose, São Paulo, Brazil) were hydrolyzed using aqueous maleic acid solution of 50 wt-% at 90 °C for 60 min. After separating cellulose nanocrystals through dialysis, the partially hydrolyzed cellulosic solids were fed into a pilot scale homogenizer (GEA Noro Soavi Ariete NS3015, GEA, Düsseldorf, Germany) for mechanical fibrillation. The resultant CNF were obtained after 5 passes through the homogenizer [35] (Lot BCNF_M50T90t60, 0.75% wt., pH 7.03, charge density 337.77 ± 62.67 µeq/g); β-cyclodextrin (>98%, CD) was purchased from Tokyo Chemical Industry America, epichlorohydrin (99%, EPI) was purchased from Acros Organics; microcystin-LR (>95%, MC) was purchased from Cayman Chemicals, polyvinyl sulfuric acid potassium salt (0.001 N, PVSK) and polydimethyl diallyl ammonium chloride (0.001 N, pDADMAC) were bought from BTG, and sodium hydroxide (50% *w*/*w*) was purchased from J.T. Baker. The water used was deionized and purified with a Thermo Scientific Barnstead Nanopure (18.2 MΩ cm). Fifty mM Tris-HCl, pH 7.4 buffer was used for dissolving microcystin where required, in concentrations of 0.5 µg/mL. Polyethyleneimine (PEI) and all other polymeric solutions were utilized at a concentration of 0.1% (*m*/*v*) in ultrapure water.

### 2.2. Cellulose Nanofibril Characterization

#### 2.2.1. Dry Content Determination

The dry content was determined following the TAPPI standard T550 [37]: Nanocellulose suspensions were weighted in aluminum pans and dried overnight in an oven at 105 °C. The dry material was weighted and subtracted from the moisture weight. Consequently, the percentage was calculated based on the moisture content (*MC*%) according to Equation (1).

(1) $$MC\%=\frac{mass_{wet}-mass_{dry}}{mass_{wet}}*100\%$$

The average and standard deviation of three replicates was calculated and the dry content was determined as the difference of the 100% and the *MC*%.

#### 2.2.2. Charge Density and pH Measurements

The charge density of the CNF was measured using a method adapted from Espinosa et al. [38], with measurements repeated three times and then averaged. Briefly, CNF suspensions were prepared at 0.04 wt-% and sonicated for 10 min using a Vibra Cell sonicator (Sonics & Materials, Inc Newtown, CA, USA), with 20 KW and 25% of amplitude, to effectively disperse the fibrils. Then, 25 mL of polydimethyl diallyl ammonium chloride (pDADMAC) was added to 15 mL of CNF suspension and mixed for 10 min, followed by centrifugation at 3000 rpm for 15 min. After centrifugation, 10 mL of the supernatant was analyzed in a Laboratory Charge Analyzer Chemtrac LCA-1, (Norcross, GA, USA). Next, a titration using polyvinyl sulfuric acid potassium salt (PSVK) was performed, stopping the equipment until it reached a streaming current value (SCV) of zero. The volume of anionic titrant consumed was used for the final calculations using Equation 2.

(2) $$Charge\;density=\frac{\left(\left[pDADMAC\right]*V_{p-DADMAC}\right)-\left(\left[PVSK\right]*V_{PVSK}\right)}{W_{dry\;CNF\;sample}}$$

where [pDADMAC] is the concentration of the cationic polymer, $V_{pDADMAC}$ is the used volume of p-DADMAC, [PVSK] is the concentration of the anionic titrant, $V_{PVSK}$ is the consumed volume of titrant, and $W_{dry\;LCNF\;sample}$ is the weight of the dry CNF sample.

The CNF suspensions were used as received, pH was measured using a SympHony Benchtop Multi Parameter Meter B30PCI (VWR^®^) equipped with pH and conductivity electrodes. Measurements were repeated 15 times and the average and standard deviation were reported.

### 2.3. Polymerization of β-Cyclodextrin

Five grams of β-cyclodextrin was dissolved in 40 mL of 20% NaOH solution. Next, the temperature was raised to 65 °C while 13.8 mL (16.3 g) of epichlorohydrin (≈0.8 mL/min) was added dropwise using an addition funnel. The reaction was stopped after 2 h by adding 150 mL of acetone. Once room temperature was reached, the solution was vacuum filtered through a Whatman ashless glass filter paper No 1 and placed in a 100 mL extraction thimble, which was placed into a Soxhlet extractor. The extraction of residual epichlorohydrin was carried out with 150 mL of acetone. After 12 cycles (ca. 3 h) at 80 °C, the solid poly(β-cyclodextrin) (PCD) was recovered and ground using a mortar and pestle and kept in a desiccator for further use.

### 2.4. Preparation of CNF/β-Cyclodextrin Co-Polymers

#### 2.4.1. Synthesis of CNF-CD

A CNF suspension of 0.4 wt-% was stirred at 4 °C for 20 h. Then, the suspension was transferred to a three-neck round bottom flask and 30 mL of NaOH (40%) was added at room temperature to activate the CNF hydroxyl groups. After this, 5 g of β-cyclodextrin was added to the mixture and stirred for 1 h. The temperature was raised to 65 °C while adding dropwise 15.2 mL (18 g) of epichlorohydrin (≈0.8 mL/min) with an addition funnel. The reaction was carried out for 2 h at 65 °C under stirring at 180 rpm. After cooling down, the solution was filtered under vacuum using PYREX™ Buchner funnel with a fritted disc of pore size 4–5.5 µm until the no more water was released.

The filtered material was placed into an extraction cellulose thimble in a Soxhlet extractor and washed with 150 mL of acetone at 80 °C for 16 cycles (ca. 16 min/cycle). The washed material was resuspended in 150 mL of ultrapure water and neutralized using 1 M HCl solution. The neutralized co-polymer was filtered under vacuum and stored at 4 °C.

#### 2.4.2. Synthesis of CNF-PCD

The same procedure described above was followed for the synthesis of CNF/poly(β-cyclodextrin) co-polymer. However, in this synthesis 5 g of the poly(β-cyclodextrin) was used instead of pristine β-cyclodextrin.

### 2.5. Characterization Techniques

#### 2.5.1. Fourier-Transform Infrared Spectroscopy with Attenuated Total Reflectance (FTIR-ATR)

To assess the modification of the CNF fibers, dry samples were analyzed using a Perkin Elmer Spotlight 400 FT-IR Imaging System (Waltham, MA, USA) with an ATR accessory with diamond/ZnSe crystal and a resolution of 4 cm^−1^ to reveal surface modification. First, a background spectrum was collected before each set of measurement with the same number of scans. To achieve a high spectral resolution, 1024 scans per spectrum were performed. The analysis of the data was with Spectrum 6 Spectroscopy Software (PerkinElmer, Waltham, MA, USA).

#### 2.5.2. X-Ray Photoelectron Spectroscopy (XPS)

Samples were mounted on an XPS sample holder using UHV compatible carbon tape and pre-evacuated overnight together with a piece of pure cellulose as the in-situ reference monitoring analysis conditions during the measurement [39]. The gathered data were recorded using monochromatic Al Kα irradiation at only 100W and under neutralization. In data analysis, high-resolution C 1s regions were fitted with Gaussian components of equal half widths, and the binding energies of all spectra were charge-corrected using the main component of cellulose, namely C–O at 286.7 eV, as the BE reference [40].

#### 2.5.3. Thermogravimetric Analysis (TGA)

Dry samples were tested in aluminum pans in a TGA-50 from Shimadzu (Kyoto, Japan). For this, samples were heated from room temperature to 600 °C at a rate of 10 °C/min under a nitrogen atmosphere and the data was processed with TA60 software version 2.11 from Shimadzu.

#### 2.5.4. Atomic Force Microscopy (AFM)

Morphological characterization of the nanofibrils in drop-cast and the surfaces from QCM-D post MC adsorption were observed using a AFM Dimension 3100 by Bunker (formerly Digital Instruments, Veeco, Plainview, NY, USA). The presented topographical images were obtained by tapping mode using a velocity in amplitude of 2.35 Hz using a Nano World (Innovative Technologies, Richmond, VA, USA) FM 20 silicon SPM-sensor cantilever at resonance frequency of 75 kHz and force constant of 2.8 N/m; scan sizes were of 5 × 5 µm and 1 × 1 µm. Processing of the images and roughness calculations were made by Gwyddion software 2.49 (SourceForge).

For Spin coated surfaces, A 20 mL of CNF or derived suspension (0.01% w/w in ultrapure water) were placed in an ice bath and sonicated during 5 minutes at 25% amplitude in a Branson Digital Sonifier 450 (Branson Ultrasonics Corporation, 230 V, 50/60 Hz, Danbury, CT, USA) with 13 mm solid extenders, using 3 s sonication and 2 s standby program. Subsequently, the samples were centrifuged at 10,000 rpm during 40 min using mini-spin Eppendorf centrifuge. The topography images were obtained with a NanoScope V controller (Dimension Icon, Bruker Corporation, Billerica, MA, USA), operating in tapping mode. Three scanning areas in all samples were selected: 5 μm × 5 μm, 3 µm × 3 µm, and 1 µm × 1 µm. Furthermore, the image correction applied was flattened during image analysis (NanoScope 8.15 software, Bruker, Billerica, MA, USA).

### 2.6. Quartz Crystal Microbalance with Dissipation Monitoring (QCM-D)

In situ formation of CNF, CNF-CD, and CNF-PCD surfaces on gold sensors and the adsorption of microcystin-LR (MC) were studied with a QSense Analyzer from Biolin Scientific (Västra Frölunda, Västra Frölunda, Sweden). The basic principle of the QCM-D is the following: Changes in frequency (Hz) of a piezoelectric sensor that has a base resonance of 5 MHz and its overtones 15, 25, 35, 45, 55, and 75 MHz are monitored. These changes in frequency resonance are proportional to a change in mass on the sensor, as only the surface is interacting with a flow of matter, then those changes are correlated to the mass adsorption on the sensor surface [41,42,43]. Moreover, Sauerbrey determined this relationship for rigid layers (D > 0) under uniform mass distribution on the surface and when the mass is much less than the mass of the crystal:

(3) $$\Delta m=C*\Delta f*n^{-1}$$

where, *C* is −17.7 ng/cm^2^ and is a constant value for 5 MHz crystals, Δ*f* is the change of frequency, and n is the overtone number. However, most of the polymeric systems do not generate a rigid layer to result in underestimation of the adsorbed mass using Equation 3. Some other models have been developed to address the change in mass, such as Voigt’s and Maxwell’s that considered the density, and dynamic and static viscosity of the adsorbed materials, as well as the crystals [44].

The dissipation factor is related to the viscoelastic properties of the layers formed on the crystal, as it translates to the relationship of energy dissipation from the sensor to the fluid and the energy stored (Equation 4). It can be measured as inversely proportional to the decay time constant (τ) and the resonant frequency (*f*) as shown in Equation 5 [44].

(4) $$D=\frac{E_{dissipated}}{2\pi E_{stored}}$$

(5) $$D=\frac{1}{\pi f\tau }$$

If the generated film is viscoelastic, energy would be dissipated due to the oscillation of the layer and thus more energy will be lost because of longer decay times, i.e., when more energy is stored, as the surface becomes more rigid, a decrease in the D-factor will be observed [45].

To obtain information about the behavior of the surface and the adsorption of microcystin, measurements were carried out at 25 °C with a constant flow of 150 µL/min on gold coated crystals using PEI as anchoring solution. Previously, crystal sensors were placed in Novascan PSD Series Digital UV Ozone System (Boone, IA, USA) for 15 min to eliminate dust and activate the surface. The active surfaces were generated in situ by flowing the polymer solution (0.1% (*m*/*v*)) until stabilization was achieved, followed by adsorption of microcystin-LR (0.5 µg/mL) at the same flow rate. Accordingly, the data was collected at multiple frequencies, however only the third overtone is presented on sensogram to facilitate discussion and interpretation. The adsorbed mass was calculated using the Broadfit model (based on the Voight model) with dissipation dependency from frequency changes, using DFind Software from Biolin Scientific (Västra Frölunda, Sweden). For this, literature densities were used for CNF and microcystin-LR (1.20 g/cm^3^ and 1.299 g/cm^3^), while for the modified polymers, it was calculated by compacting dry material into 1 mL containers and weighing, coming to values of 1.096 and 0.954 g/cm^3^ for CNF-CD and CNF-PCD, respectively.

## 3. Results and Discussions

### 3.1. Characterization of CNF and the Modified Polysaccharides

#### 3.1.1. CNF Suspensions 

The CNF suspension was characterized prior to use and after its modification with β-cyclodextrin. Solid content of the white milky suspension was determined to be 0.75 wt.%. The suspension pH and charge density were 7.0 µeq/g and 337.7 ± 62.6 µeq/g, respectively. The latter is due to the carboxylation at cellulose C6 position through esterification by hydrolysis using concentrated Maleic acid [35].

#### 3.1.2. Synthesis of Poly(β-Cyclodextrin) (PCD)

**Figure 4:**
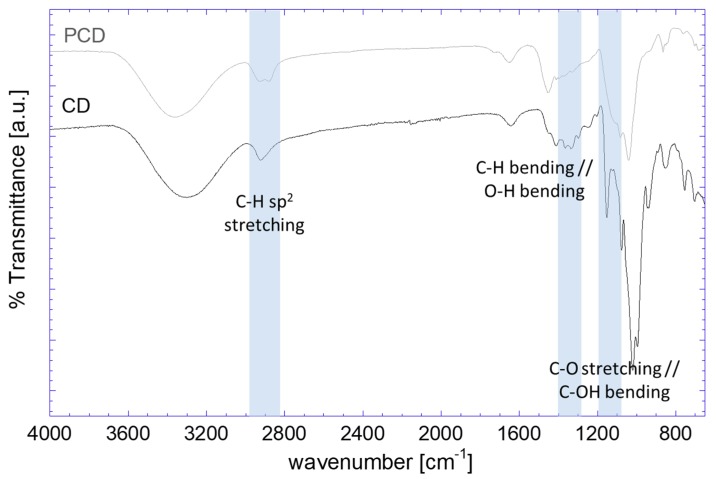
FTIR-ATR spectra of cyclodextrin and poly (cyclodextrin) obtained by crosslinking with epichlorohydrin.

The crosslinking of the CD with EPI resulted in a granular texture after vacuum filtration. To assess the reaction, FTIR was also performed as shown in Figure 4, where the spectra of the CD and PCD are presented. The main differences caused by EPI linkage resulted in spectral changes in the C–H stretching band (2900 cm^−1^). The presence of ether bonds was clearly reflected from the significant reduction in the C–O signal (1200 cm^−1^). Furthermore, the variations between 1400 cm^−1^ and 1300 cm^−1^ are mainly due to changes in the bending modes of C–H and in-plane O–H, which may point towards more diverse movement that these groups can undergo through the bridges formed by EPI. It should be noted here that the observed spectral characteristics are in agreement with those reported in the literature for crosslinking of cyclodextrin with epichlorohydrin [46].

#### 3.1.3. Synthesis of CNF-CD and CNF-PCD

The first indication of successful grafting of the CD and PCD to the CNF was given by the appearance and texture of each system. Observable differences in the physical state between the different products were seen, from a fine powder for CD to a more flake-like of the crosslinked PCD, up to gels from the CNF-CD and CNF. Both the CNF-CD and CNF-PCD sample had a solid content of 22 wt.%. However, CNF-PCD appeared more solid-like than CNF-CD, which looked more like a gel.

The charge density was reduced to 128.8 ± 10.1 µeq/g for the CNF-CD and to 166.6 ± 24.0 µeq/g for the CNF-PCD. The reduction in charge densities of modified CNFs was likely caused by the reactions of carboxyl and hydroxyl groups with EPI and with the CD or PCD. Moreover, the charge density difference between the CNF-CD and CNF-PCD might be related to the higher amount of hydroxyl groups that were added with the PCD and with the EPI linkages than obtained by the reaction with the pristine CD. However, the difference could also be a consequence of a lower efficiency in the reaction with PCD, resulting in less reacted sites in the fibrils.

**Figure 5:**
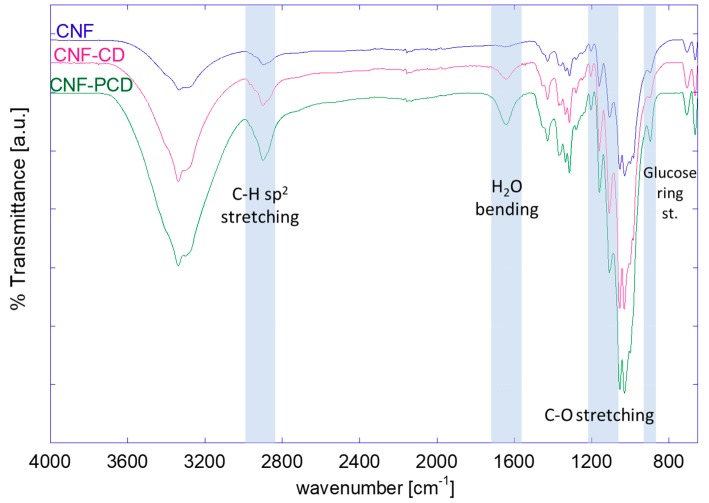
FTIR-ATR spectra comparing cellulose nanofibrils (CNF) and modified cellulose nanofibrils (CNF-CD and CNF-PCD).

The modification of the CNF was then confirmed through FTIR analysis. In Figure 5, the spectra of the modified CNFs are shown. The main changes between them are in the intensity of the bands of C–H stretching (2900 cm^−1^) and the increase in intensity related to the glucose ring out-of-phase stretching (890 cm^−1^), which can also be seen to be more pronounced in the system with PCD as more anhydroglucose units are present. Similarly, we also see an increase in H_2_–O bending (1650 cm^−1^) related to adsorbed water in the structures. Finally, the definition of the bands related to C–C and C–OH stretching (1020 cm^−1^) confirm the etherification of the materials, as well as the increase of secondary alcohols from either epoxy derivatives or CD.

**Figure 6:**
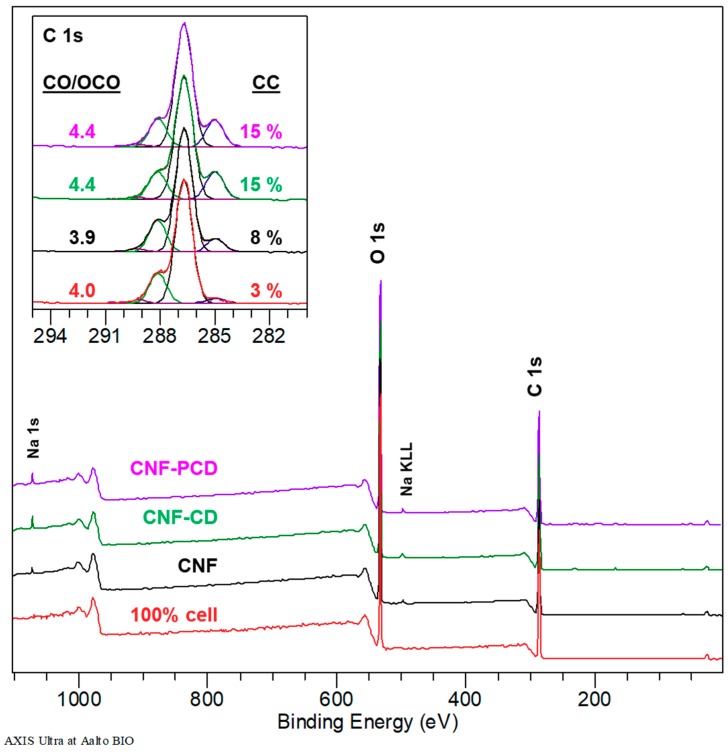
XPS wide energy region spectra comparing a pure cellulose reference, cellulose nanofibrils (CNF) and modified cellulose nanofibrils (CNF-CD and CNF-PCD). The insert shows the high-resolution C 1s spectra.

Similarly, the modification can be observed in Figure 6 where the XPS wide energy region spectra of the three materials (CNF, CNF-CD, and CNF-PCD) are shown as well as a reference of 100% pure cellulose. The main differences were observed in the C 1s high resolution spectra, where the ratio of CO/OCO increased from 3.9 to 4.4 as a result the modification. This indicates the presence of other surface elements besides anydroglucose monomeric units from cellulose. The increase in the CC component, i.e., carbons without oxygen neighbors, is typical to CNF, and most possibly related to the ultra-high vacuum conditions of XPS [47]. It is further increased in the modified materials, which could be linked with the presence of the EPI linkages.

**Figure 7:**
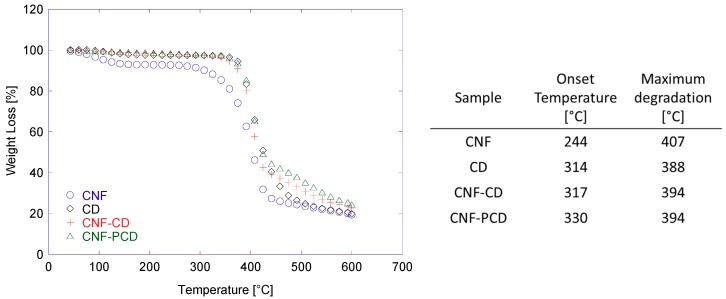
Thermogravimetric analysis of the CNF, CD, and the modified cellulose nanofibrils (CNF-CD and CNF-PCD). The thermogram (**left**) shows the behavior of the material, and the table (**right**) presents the main extracted data for each sample.

TGA data also suggested the successful modifications of the CNF, as the onset degradation temperatures were increased, when compared to that of the unmodified CNF (Figure 7). As expected, CNF-PCD was the most stable as it possesses a higher crosslinking density, i.e., the onset degradation temperature of CNF-PCD was 13 °C higher than that of CNF-CD. When the first derivative was observed, there was no significant difference in the maximums of both products (Figure S1). However, they are set in between the CNF and CD with a single peak which can be considered indicative for a successful modification. Furthermore, a second change in slope from 450 °C to 554 °C for CNF-CD and from 450 °C to 585 °C for CNF-PCD was observed, both of which can be related to a bridge formed by EPI.

### 3.2. Formation of Surfaces in Situ on QCM-D and Adsorption of Microcystin-LR

**Figure 8:**
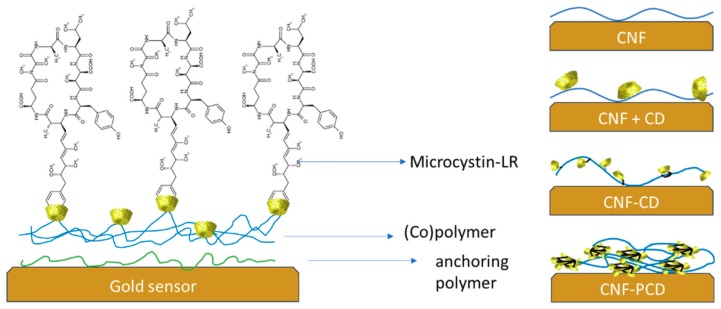
Scheme of the sequence used in the QCM-D to monitor the adsorption of the microcystinon the generated (co)polymer layers.

To test the adsorption capacity of the modified CNF in capturing microcystin-LR, surfaces were generated in situ on the QCM-D by flowing the substrates and once stabilized, flowing the toxin. Two media; water and TRIS-buffer; were used in order to compare the changes in the adsorption of MC under different ionic concentrations. A simplified scheme of the layers used is presented in Figure 8. PEI was used as an anchoring polymer (first layer) in which the (co)polymers CNF, CNF-CD, or CNF-PCD were then adsorbed. Consequently, a layer-by-layer (CNF + CD) approach was also studied to assure that the capture of the microcystin was due to its chemical bonding with β-cyclodextrin instead of merely physical adsorption on the unmodified CNF surface or to the surface of the CD, independent of the availability of the hydrophobic cavity that can be blocked by H-bonding with the CNF fibrils.

**Figure 9:**
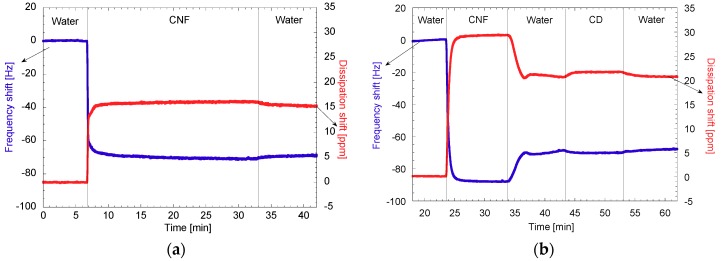

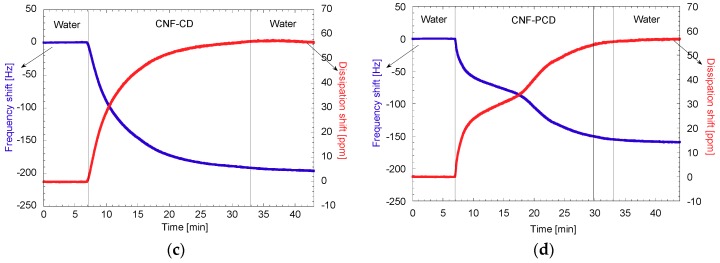
QCM-D sensograms of the in situ surface generation from (**a**) CNF, (**b**) CNF and flushed CD (CNF+CD), (**c**) CNF-CD, and (**d**) CNF-PCD. CNF, cellulose nanofibrils; CD, cyclodextrin; CNF-CD, cellulose nanofibrils grafted with cyclodextrin; CNF-PCD, cellulose nanofibrils grafted with poly-cyclodextrin.

QCM-D sensograms of the studied systems were split to facilitated discussion, with the first part being the surface generation and the second, the microcystin adsorption. The first part of the sensograms (in situ surface generation) is shown in Figure 9 and is discussed with the AFM images. For comparison, the surfaces were also generated by using spin coating and drop casting techniques.

#### 3.2.1. Surface Generation and Characterization

As mentioned, the first part of the sensograms is related to the generation of the surface in situ on the QCM-D. The formation of CNF surfaces was done twice, with the first being as a control without any modification of the surface (Figure 9a), and the second is including the addition of pure CD (Figure 9b). When the changes in frequency were observed, both CNF surfaces had a negative shift of −69 Hz and changes in dissipation between 15 ppm and 20 ppm in all repetitions, showing good consistency in the approach. When the adsorption of the CD on the CNF surface was completed (Figure 9b), the change in frequency only amounted to −2 Hz, which is not considered representative in rigid surfaces. However, the change in dissipation was more than 0.5 ppm, suggesting a change in the viscoelasticity of the surface showing there was some molecular adsorption of the CD.

For the adsorption of CNF-CD and CNF-PCD, larger changes in frequency and dissipation were observed (Figure 9c,d). Herein, the frequency shifts were −195 HZ and −158 Hz and dissipation was increased by 55 ppm and 56 ppm for CNF-CD and CNF-PCD, respectively. Both materials thus showed viscoelastic behavior. It is worth noting that the frequency and dissipation shifts were more significant than in the case of unmodified CNF. These differences in the frequency and dissipation shifts were almost three times higher for both of the modified materials than for the pristine CNF; these differences can be related to the difference in viscosity of the modified materials as well as improved interactions with the amino groups of the anchoring polymer. The changes in dissipation can be associated with the water uptake which is known to occur in highly hydrated fibrillar systems [48,49].

**Figure 10:**
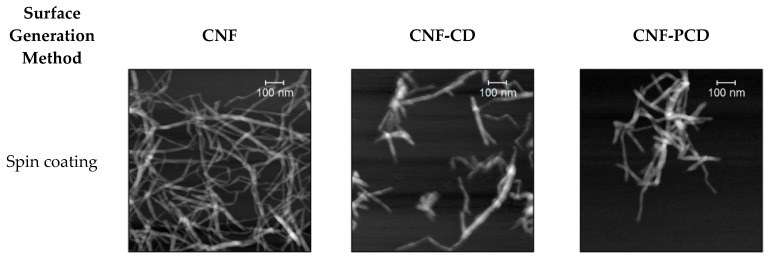

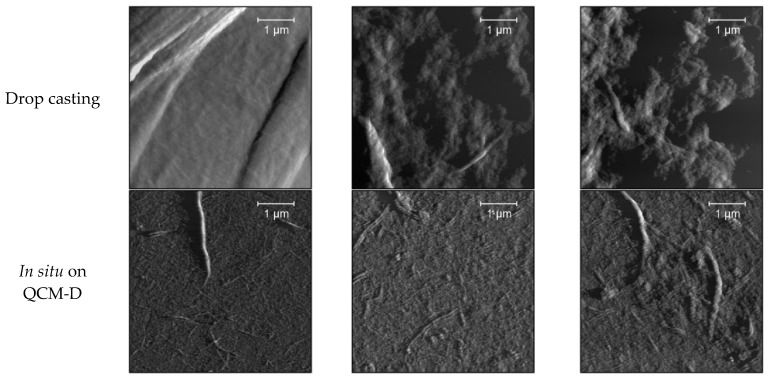
AFM images of the cellulose nanofibrils (CNF) and the modified cellulose nanofibrils (CNF-CD and CNF-PCD) and the average rugosity (Ra), each row was generated by a different technique, while the last one was measured after adsorption of microcystin in the QCM-D.

AFM images of the different surfaces are shown in Figure 10. After spin coating, the CNF films showed well-dispersed fibril networks, which are not observed for CD or PCD modified fibrils. In the case of modified fibrils, agglomeration was visible, as well as higher points or mounds along the surface, therefore this can be an indication of binding from the CD and the fibrils. Furthermore, when the average rugosity (Ra) of the images is compared, the CNF-PCD image is approximately two times of Ra for the CNF-CD image. This confirms a higher grafting density for CNF-PCD which is in agreement with the higher charge density and the higher dissipation shift from QCM-D experiments.

The CNF surface had a smoother appearance when compared to the other materials. However, it is visible that microfibers and finer nanofibrils are present in all. While comparing the CNF-CD and CNF-PCD, wider fibrils are found on the surface of CNF-PCD Moreover, the AFM images of CNF-CD and CNF-PCD show some larger particles which is likely to be due to the presence of the CD on the surface.

Table 1 compares the roughnesses between the surfaces of CNF, CNF-CD, and CNF-PCD generated by the three methods (spin-coating, drop-casting, and in-situ adsorption in the QCM-D). One can observe that the roughness increases with the increased grafting on the surface. However, it is worth mentioning here that the spin-coated surfaces are not fully covered, which made the surface roughness data less comparable, as as it is virtually impossible to accurately correct for uncoated areas. However, including this skew in the data, Ra and Rq for CNF-PCD is significantly higher than CNF-CD and CNF.

Drop-casting produced thicker surfaces as more material was deposited.The rugosity difference between CNF and CNF-CD was less than 1 nm. However, the roughness of CNF-PCD surface was higher, indicating the apparent relationship between the grafting density and surface rugosity. Finally, a similar behavior was conserved even after the adsorption of MC.

#### 3.2.2. Capture of MC Followed by QCM-D

**Figure 11:**
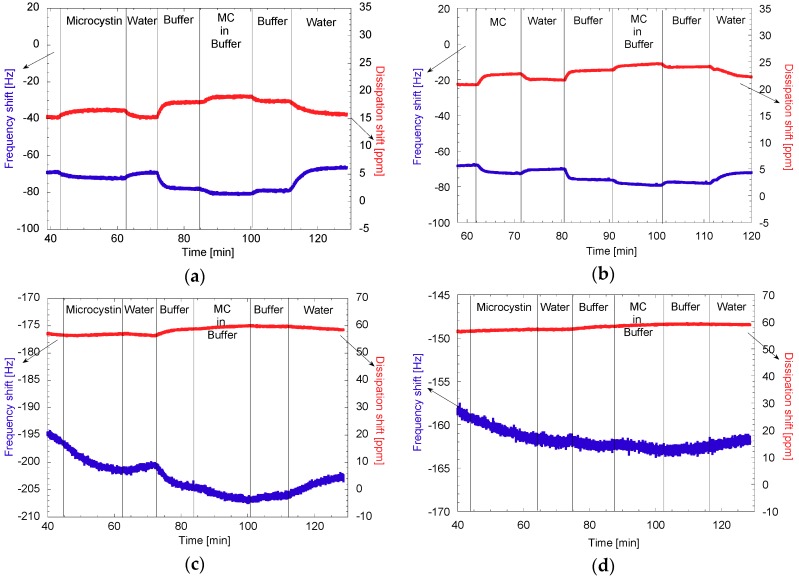
QCM-D sensograms of the adsorption of microcystin in water and Tris-HCl buffer from (**a**) CNF, (**b**) CNF and flushed CD (CNF + CD), (**c**) CNF-CD, and (**d**) CNF-PCD. CNF, cellulose nanofibrils; CD, cyclodextrin; CNF-CD, cellulose nanofibrils grafted with cyclodextrin; CNF-PCD, cellulose nanofibrils grafted with poly-cyclodextrin.

In the second part of the sensograms shown in Figure 11, the adsorption of MC was monitored first with water as a medium, followed by an adsorption in hypersaline conditions. In Figure 11a, the adsorption of MC on the unmodified surface of CNF seemed to be reversible, as the frequency level decreased to −72 Hz and went up again to −69 Hz upon rinsing with ultrapure water. When the adsorption was done in hypersaline conditions, however, a frequency shift of −2 Hz was sustained after rinsing, suggesting that this condition helped the adsorption. The adsorption in hypersaline conditions is likely due to entropic effects. Consequently, this would be due to a more energetically convenient state by having the MC on the surface than having ions from the salts, thus hurdling desorption.

In Figure 11b, the adsorption of the CD was first tested with no apparent adsorption, but when the MC was flown through the QCM-D, a frequency decrease of 2 Hz as well as an increase in the dissipation shift was observed, indicating adsorption of MC on the surface. Under hypersaline conditions, there was also a decrease in frequency, however this was smaller than the first one, probably indicating less available free space and less entropic effects as the surface energy was lowered with the adsorption of the CD.

For CNF-CD as shown in Figure 11c, the adsorption that can be seen from the decrease in the frequency shift, −5 Hz in water and −2 Hz under hypersaline conditions. Once more, showing extra adsorption when the salts were present, which can also relate to the exchange of salts for MC and the facilitating of contact with less available active spaces. The increased adsorption under hypersaline conditions can be due to the easier contact of the hydrophobic tail to the CD as well as some other types of interactions like hydrogen bonding from the aromatic at the end of the tail and the electrons on the surface of the anhydroglucose units of cellulose or CD. To investigate the driving force for the interaction, Isothermal Titration Calorimetry experiments were also carried out. Herein, the interaction of CNF-CD and MC were measured. In these experiments, the enthalpic interactions between the CNF-CD and MC were always too low for detection, indicating that the CD-MC interaction is not driven by preferential enthalpic interactions, but are instead driven by entropy, where the water from the corona around MC and from inside the CD is released upon interaction, and decreases the Gibbs-free energy of the system.

As shown in Figure 11d, adsorption on the CNF-PCD surface had a frequency shift of −4 Hz in water and −2 Hz in in hypersaline water, both of which were lower than in the observed from using CNF-CD. These differences are likely related to the denser crosslinked CD that are present in PCD, which can block the access to the hydrophobic cavities on the surface.

#### 3.2.3. Mass Modeling from QCM-D Data

**Figure 12:**
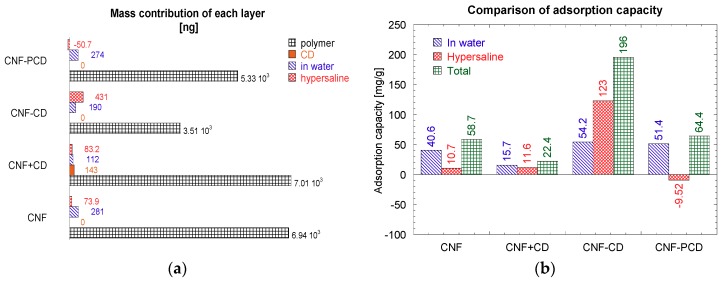
Charts of the mass obtained after the modeling the frequency and dissipation shift of the QCM-D sensograms in QSense DFind software. (**a**) Mass contribution of each layer added to the surfaces, and (**b**) microcystin-LR adsorption capability of each CNF polymer. CNF, cellulose nanofibrils; CD, cyclodextrin; CNF-CD, cellulose nanofibrils grafted with cyclodextrin; CNF-PCD, cellulose nanofibrils grafted with poly-cyclodextrin.

After modeling the data obtained from the sensograms with QSense DFind software, the mass contribution of each layer was calculated, taking into account densities, frequency, and dissipation shifts. The average masses obtained in each event are presented in Figure 12a and the ratios of MC to CNF, CNF + CD, CNF-CD, and CNF-PCD are presented in Figure 12b. The total MC mass was calculated from the frequency shift values obtained after a final rinse with ultrapure water.

When the masses of the materials on the surface were calculated and compared (Figure 12a), the CNF presented a higher mass with ca. 7 mg on both surfaces (CNF and CNF + CD). After CNF, CNF-PCD presented more adsorbed mass than CNF-CD, this latter one had a higher drop in frequency shift in Figure 9c, but also had a lower density than CNF-PCD, then the interdependency between these two parameters and the dissipation gave a mass of 3.5 mg for CNF-CD and 5.3 mg for CNF-PCD.

For CNF-CD, even when the frequency shift was minimal in the adsorption step of the CD, a mass of 143.3 ng was calculated which can explain the decrease in frequency that was observed in the MC step. This adsorption amounted to 112 ng of MC in water and an extra 83 ng in the hypersaline Tris buffer. This total mass adsorbed in water equaled 281 ng of MC adsorbed on pure CNF, which indicates that the CD competed with the MC for free space.

Overall, the adsorption increased with the presence of crosslinking, as both CNF-CD and CNF-PCD had better adsorptions than the CNF and CNF-CD. Moreover, CNF-PCD had a maximum adsorption of 64.4 mg/g, with 51.4 mg/g of it corresponding to adsorption in water. The adsorption under hypersaline conditions was negative in the step calculation, however, after the final rinse with water the final adsorption value was higher meaning that some adsorption occurred also during the hypersaline step.

From all the set of experiments, CNF-CD was the only material that presented a different trend in the adsorption values Furthermore, this was the only case in which the highest adsorption was under hypersaline conditions. This difference is likely due to a different mechanism for adsorption compared to other three, and this can only correspond to the active capturing by the hydrophobic cavity of the available CD. After the final rinse with ultrapure water, the maximum adsorption stable on the surface was 196 mg/g.

It is important to mention that even though the adsorption capacities were obtained using a non-standardized technique; the adsorption capacity of the CNF-CD is more than double that some other bio-based materials reported in the literature. For example, CS + CEL composite generated by Tran et al. [50], was reported to adsorb 96 mg/g, while a magnetic nanocomposite (G-Fe_2_O_3_-CD) had a β-cyclodextrin removal capacity of 140 mg/g [16].

## 4. Conclusions

The present work demonstrates that cellulose nanofibrils (CNF) can be modified with β-cyclodextrin to enhance the removal of microcystin-LR from aqueous solutions. Two different modification approaches were investigated and one step crosslinking onto the surface was found to be the most effective by removing up to 196 mg/g according to the modelled QCM-D measurements. This material presents an opportunity to develop novel sustainable materials that can help improve the water quality by using non-specific interaction. While the cyclodextrin hydrophobic cavity allows to adsorb hydrophobic substrates and aromatic pollutants from water sources, the nanofibrillar matrix of CNF provides an increased surface area for the adsorption. The combination of both phenomena can then be maximized when complex structures, such as membranes or aerogels, are generated from this material.

## 5. Future Work

Even though the CNF-CD was a good material to adsorb microcystin, the non-specific nature of the interactions with this material possesses a great capacity to capture other hydrophobic compounds that might be in the same water sources. Furthermore, competition between these different pollutants at diverse pH and ionic strength are phenomena that could have an impact on the performance.

One of the big challenges that was presented in this work was assessing the amount of cyclodextrin attached. This due to the similarities in chemical structure between the base materials for the composites—solid state nuclear magnetic resonance was tried but good results were not obtained. Consequently, the increase in expertise of collaborations with ionic liquid assisted NMR could lead to a better understanding of the material.

Finally, another challenge for the utilization of this material is the formation of 3-D structures that are stable enough that cycles of washing, adsorption, and desorption are possible. There is some literature on how to break complexes between microcystin and cyclodextrin, however the 3-D formation should be studied in parallel.

## Figures and Tables

**Table 1:** Roughness comparison between the systems surfaces.

System	Average Roughness (Ra) [nm]	Root Mean Square Roughness (Rq) [nm]	Average Maximum Height of the Roughness (Rz ISO) [nm]
*Spin coated*			
CNF	1.43	1.80	14.7
CNF-CD	0.84	1.25	10.9
CNF-PCD	1.95	2.46	15.0
*Drop Casted*			
CNF	2.00	3.00	13.0
CNF-CD	2.80	3.90	14.7
CNF-PCD	4.20	4.90	16.8
*In situ on QCM-D*			
CNF/MC	0.30	0.53	1.99
CNF-CD/MC	1.18	1.45	6.00
CNF-PCD/MC	2.06	2.44	9.23

## Supplementary Materials

The following are available online at https://www.mdpi.com/2073-4360/11/12/2075/s1, Figure S1: First derivate of thermogravimetric analysis of the CNF, CD, and the modified cellulose nanofibrils (CNF-CD and CNF-PCD).
