# Supplementary material for: Cellulose-Cyclodextrin Co-Polymer for the Removal of Cyanotoxins on Water Sources
Source: Polymers (Basel). 2019 Dec 12;11(12):2075. doi: 10.3390/polym11122075 (PMC6960681; doi:10.3390/polym11122075)
Supplement: Supplementary file 1 [file polymers-11-02075-s001.pdf]

*polymers*

MDPI

*Article*

# Cellulose-Cyclodextrin Co-Polymer for the Removal of Cyanotoxins on Water Sources

**Diego Gomez-Maldonado [1], Iris Beatriz Vega Erramuspe [1], Ilari Filpponen [1,2], Leena-Sisko Johansson [3], Salvatore Lombardo [4], Junyong Zhu [5], Wim Thielemans [4] and Maria S. Peresin [1,*]**

[1] Forest Products Development Center, School of Forestry and Wildlife Science, Auburn University, 520 Devall Drive, Auburn, AL 36830, USA; dzg0023@auburn.edu (D.G.-M.); ibv0002@auburn.edu (I.B.V.E.); ilari.filpponen@auburn.edu (I.F.)

[2] Department of Chemical Engineering, Alabama Center for Paper and Bioresource Engineering (AC-PABE), Auburn University, 358 Ross Hall, Auburn, AL 36849, USA

[3] Department of Bioprocesses and Biosystems, Aalto School of Chemical Technology, BIO2, P.O. Box 16100, 02150 Espoo, Finland; leena-sisko.johansson@aalto.fi

[4] Renewable Materials and Nanotechnology Research Group, Department of Chemical Engineering, KU Leuven, Campus Kulka Kortrijk, Etienne Sabbelaan 53, 8500 Kortrijk, Belgium; salvatore.lombardo@kuleuven.be (S.L.); wim.thielemans@kuleuven.be (W.T.)

[5] USDA Forest Products Laboratory, 1 Gifford Pinchot, Madison, WI 53726, USA; junyong.zhu@usda.gov

* Correspondence: soledad.peresin@auburn.edu; Tel.: +1-334-559-1143

Received: 7 November 2019; Accepted: 9 December 2019; Published: date

**Supplementary Materials:**

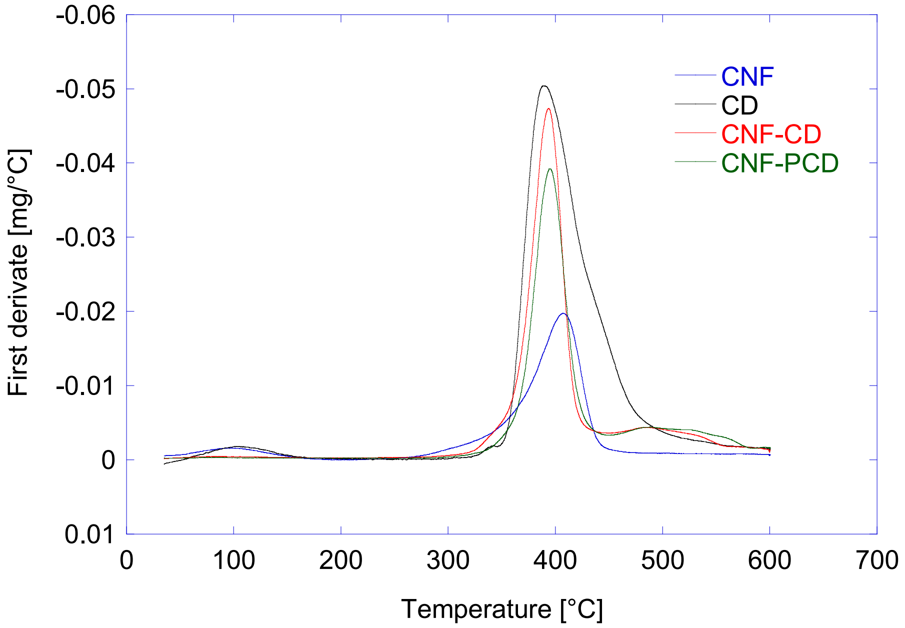


*Figure S1. First derivate of thermogravimetric analysis of the CNF, CD, and the modified cellulose nanofibrils (CNF-CD and CNF-PCD).*
